# Risk and Protective Factors of Postoperative and Persistent Hypoparathyroidism after Total Thyroidectomy in a Series of 1965 Patients

**DOI:** 10.3390/cancers16162867

**Published:** 2024-08-17

**Authors:** Silvia Dughiero, Francesca Torresan, Simona Censi, Caterina Mian, José Luis Carrillo Lizarazo, Maurizio Iacobone

**Affiliations:** 1 Endocrine Surgery Unit, Department of Surgery, Oncology and Gastroenterology, University Hospital of Padua, 35128 Padova, Italy; silvia.dughiero.1@studenti.unipd.it (S.D.); francesca.torresan@uinpd.it (F.T.);; 2 Unit of Endocrinology, Department of Medicine, University of Padua, 35128 Padova, Italy; simona.censi@unipd.it (S.C.); caterina.mian@unipd.it (C.M.)

**Keywords:** hypoparathyroidism, total thyroidectomy, lymph-node neck dissection, morbidity

## Abstract

**Simple Summary:**

Postoperative hypoparathyroidism (HypoPTH) is the most common complication following total thyroidectomy, but the literature still lacks clear risk and protective factors. This study aimed to evaluate and compare the risk factors for post-surgical HypoPTH in a large series of patients at a tertiary academic high-volume center undergoing total thyroidectomy for both benign and malignant diseases. It also focused on identifying risk and protective factors for persistent HypoPTH.

**Abstract:**

Background: Postoperative hypoparathyroidism (HypoPTH) is the most common complication following total thyroidectomy. Several risk factors have been identified, but data on postoperative follow-up are scarce. Methods: The study focused on 1965 patients undergoing surgery for benign and malignant thyroid diseases at a tertiary-level academic center. Anamnestic, biochemical, surgical, pathological, and follow-up data were evaluated. HypoPTH was defined by a serum concentration of PTH < 10 pg/mL on the first or the second post-operative day. Persistent HypoPTH was defined by the need for calcium/active vitamin D treatment > 12 months after surgery. Results: Postoperative HypoPTH occurred in 542 patients. Multivariate analysis identified the association of central lymph-nodal dissection, reduced preoperative PTH levels, a lower rate of parathyroid glands preserved in situ, and longer duration of surgery as independent risk factors. At a median follow-up of 47 months, HypoPTH regressed in 443 patients (more than 6 months after surgery in 7%) and persisted in 53 patients. Patients receiving a lower dose of calcium/active vitamin D treatment at discharge (HR 0.559; *p* < 0.001) or undergoing prolonged, tailored, and direct follow-up by the operating endocrine surgeon team had a significantly lower risk of persistent HypoPTH (2.4% compared to 32.8% for other specialists) (HR 2.563; *p* < 0.001). Conclusions: Various patient, disease, and surgeon-related risk factors may predict postoperative HypoPTH. Lower postoperative calcium/active vitamin D treatment and prolonged, tailored follow-up directly performed by operating endocrine surgeons may significantly reduce the rate of persistent HypoPTH.

## 1. Introduction

Post-surgical hypoparathyroidism (HypoPTH) is the most frequent morbidity after total thyroidectomy, with a prevalence of 1.2–40% for the transient variant (with recovery of parathyroid function within 6 months after surgery) and up to 33% for the permanent variant [1,2,3,4,5].

However, the definition itself of post-surgical HypoPTH is heterogeneous and still debated; indeed, it has been defined according to low postoperative serum parathormone (PTH) or calcium levels (with various thresholds); the need for calcium/active vitamin D supplementation; and the presence of symptoms related to hypocalcemia [4,6].

As a consequence, the reported risk factors for HypoPTH are inhomogeneous and fairly discordant. Female gender [7,8,9,10,11], advanced age at the time of surgery [12,13,14,15], autoimmune thyroid diseases [16], surgery for thyroid malignancy and lymph-nodal dissection [17,18], reoperations, accidental parathyroid removal [19,20], and surgical expertise [21,22] are the most frequently reported.

The identification of reliable markers of HypoPTH is essential to start preventive treatment, allowing a safe and possibly early hospital discharge before patients become symptomatic, since hypocalcemia may occur even very late (up to 64 h after surgery) [4].

Moreover, it is relevant to identify (and possibly eliminate) risk factors for definitive HypoPTH and differentiate patients with transient postoperative parathyroid insufficiency from those with protracted and permanent HypoPTH that need a different follow-up and have different clinical consequences [23,24].

Unfortunately, most patients are lost to prolonged follow-up, making any conclusion about the rate and risk factors for persistent and definitive HypoPTH strongly debatable.

This study aimed to evaluate and compare the risk factors for post-surgical HypoPTH in a large series of patients at a tertiary academic, high-volume center, also focusing on the risk and protective factors for persistent HypoPTH.

## 2. Materials and Methods

The present study focused on a series of 1965 consecutive patients undergoing total thyroidectomy for benign and malignant thyroid diseases at the Endocrine Surgery Unit of Padua University Hospital, Italy, from January 2016 to September 2021. Surgery was performed directly or under the guidance of experienced and high-volume endocrine surgeons. Surgery was indicated in the case of benign involvement because of the presence of large bilateral symptomatic goiters or refractory hyperthyroidism or because of preoperative suspicion of malignancy according to the results of fine needle aspiration cytology.

Inpatient and outpatient medical records were retrospectively reviewed, focusing on demographic, anamnestic, biochemical, surgical, and pathological data; whenever necessary, follow-up data were finally further obtained and updated at the beginning of 2023 by phone interview with the patient and/or by the general practitioner/referral physician.

Patients taking calcium or active vitamin D analogues for any reason before surgery, with coexistent or previously operated hyperparathyroidism, or with previous neck surgery performed at other institutions were not included in the present series.

### 2.1. Demographic, Anamnestic, and Biochemical Data

Age at surgery, gender, body mass index (BMI), arterial hypertension, diabetes, and glucose intolerance were assessed. Hyperthyroidism was defined by the need for therapy with antithyroid drugs or serum high free thyroid hormones with low TSH levels at the time of surgery. The presence of laboratory autoimmune thyroiditis was assessed according to the presence of increased serum anti-thyroid antibodies in preoperative laboratory tests. Preoperative serum total calcium (corrected according to the value of serum albumin) (normal values 2.10–2.55 mmol/L), phosphorus (normal values 0.87–1.45 mmol/L), vitamin D, and PTH levels were assessed. Vitamin D levels were considered normal or decreased at a cut-off of 75 nmol/L. Preoperative PTH levels were normalized and scored as a ratio (PTH value/upper limit of the normal range value for each method), since different and not comparable methods of measurement were used.

Postoperatively, serum ionized and total albumin-corrected calcium, phosphorus, and PTH levels were measured on the first and second postoperative days (POD). Postoperative PTH was analyzed in all patients using a third-generation CLIA DiaSorin Liaison (Stillwater, MN, USA) method (normal values 10–36.8 pg/mL).

### 2.2. Surgical Data

Duration of surgery, the association of lymph-nodal neck dissection to total thyroidectomy for a malignant pre-operative cytology, the eventual parathyroid reimplantation, and the number of parathyroid glands remained in situ (PGRIS) were assessed. PGRIS was calculated as follows: 4-(glands autografted + glands in the surgical specimen), as previously reported [25], and categorized as ≤2 or >2. Reoperations were defined by iterative surgery at least in the central compartment in a previously thyroidectomized patient, usually including a lymph-nodal excision for tumor recurrence.

Nodal dissection was performed in the case of malignant disease, when a nodal involvement was suspected or diagnosed pre- or intraoperatively according to cytological or frozen section examination, and systematically in all patients with medullary thyroid carcinoma. Parathyroid reimplantation was performed in the case of inadvertently removed glands (when recognized on the surface of the surgical thyroid specimen at the end of surgery), or when parathyroids could not be preserved in situ with adequate vascular supply.

### 2.3. Pathological Data

The final diagnosis (categorized as benign or malignant) and the presence of histological autoimmune thyroiditis were assessed according to the definitive pathology report; the weight of the surgical thyroid specimen was also measured.

Thyroid malignancies included papillary thyroid cancer, follicular thyroid cancer, oncocytic thyroid cancer, well-differentiated thyroid cancer not otherwise specified; poorly differentiated and anaplastic thyroid carcinomas, and medullary thyroid cancer.

### 2.4. Follow-Up Data

Most HypoPTH patients were discharged with oral calcium + active vitamin D therapy, categorized as low- (calcium ≤ 1 g/day + calcitriol ≤ 0.5 mcg/day), medium- (calcium 1–3 g/day + calcitriol 0.5–1.5 mcg/day), or high-dose treatment (calcium ≥ 3 g/day + calcitriol ≥ 1.5 mcg /day). The administration of intravenous calcium on the first or second POD was also assessed.

HypoPTH patients underwent postoperative follow-up directly by the endocrine surgical team in an outpatient clinic with a dedicated protocol including a systematic, prolonged, and tailored monitoring of serum total calcium, serum phosphorus, and PTH levels (performed at two weeks, one month, four months, and six months after surgery and then every six months), aimed at tapering and possibly discontinuing the calcium–vitamin D treatment up to the eventual recovery of both serum calcium and PTH levels (Group A) or by the general practitioner and/or other referral physicians (Group B).

The need for postoperative treatment for thyroid malignancies with radioactive iodine (radioiodine therapy) was also assessed.

Postoperative HypoPTH was defined as PTH values ≤ 10 pg/mL as measured on the first or second POD, as previously reported [4].

Persistent HypoPTH was defined by the need for calcium-active vitamin D analogues supplementation to maintain normal serum calcium levels [23] at the last available follow-up (> 1 year); conversely, regression from HypoPTH was defined by normal and appropriate calcium and PTH levels being reached without the need for any supplementation. 

Patients dropped out at the follow-up were excluded from the analysis of persistent HypoPTH due to missing data.

### 2.5. Statistics

Data were expressed as absolute numbers, percentages, means, medians, standard deviations (SD), and ranges. Statistical analysis was performed using R^®^ version 4.2.0 (R Development Core Team, Vienna, Austria). Continuous variables were tested by Kolmogorov–Smirnov test, and if the distribution was normal, a two-sided *t*-test was used, and the data were assessed as mean; otherwise, a Mann–Whitney U-test was used, and the data were expressed as median. Discrete variables were analyzed with Fisher’s exact test. The analyses for transient HypoPTH were performed using univariate and multivariate logistic regression with stepwise selection based on *p*-values for the multivariate analysis. Cox proportional hazard regression analyses were used to analyze the factors for persistent HypoPTH considering the duration of the condition in months, with stepwise selection based on backward *p*-values (threshold 0.05) for the multivariate analysis. Kaplan–Meier and log-rank tests were used to set the probability of cure in patients with persistent HypoPTH. Values of *p* < 0.05 were considered significant.

## 3. Results

The results are summarized in Table 1. The median age in the entire population was 54 (range 6–86); the female/male ratio was 3.44. Arterial hypertension was present in 28.1%; diabetes or glucose intolerance in 6.4%; hyperthyroidism in 25.6%; laboratory and histological autoimmune thyroiditis in 39% and 18.2%, respectively; malignant disease at final histology in 42.9%; and lymph-nodal dissection was performed in 25.6%.

Postsurgical HypoPTH occurred in 542 patients (27.6%).

### 3.1. Risk and Protective Factor of Immediate HypoPTH

At univariate analysis, HypoPTH patients were significantly younger (50.6 vs. 53.1 years; *p* = 0.001), had lower preoperative PTH ratio (0.65 vs. 0.77; *p* < 0.001), longer duration of surgery (80 vs. 75 min; *p* = 0.002), more frequently underwent a lymph-nodal dissection (37.2% vs. 24.3%; *p* < 0.001), had lower PGRIS (≤2; 68.9% vs. 26.4%; *p* < 0.001), a higher parathyroid reimplantation rate (46.7% vs. 26.2%; *p* < 0.001), and a higher rate of malignancy (30.8% vs. 25.1%; *p* = 0.005) and thyroiditis at pathology (31.5% vs. 26.2%; *p* = 0.002), while no significant differences were found for gender, arterial hypertension, diabetes and glucose intolerance, preoperative vitamin D and serum calcium levels, hyperthyroidism, laboratory autoimmune thyroiditis, reoperations, and thyroid weight.

At stepwise analysis, only lower PGRIS (OR: 5.050; *p* < 0.001); reduced preoperative PTH levels (OR: 3.190; *p* < 0.001), associated lymph-nodal dissection (OR: 1.550; *p* = 0.015), and longer duration of surgery (OR: 1.010; *p* = 0.032) were independent predictive risk factors for HypoPTH development (Table 1).

### 3.2. Risk and Protective Factors of Permanent HypoPTH 

At prolonged follow-up (median 47, range 12–80 months), 46 patients were lost to follow-up and excluded from further analyses. In the 496 remaining patients, HypoPTH regressed in 443 patients who discontinued any calcium/active vitamin D supplementation and reached normal calcium and PTH levels, while persistent HypoPTH was assessed in 53 patients (10.7% of HypoPTH patients and 2.7% of the entire series) (Table 2). At univariate analysis, patients with persistent HypoPTH had a longer duration of surgery (85 vs. 80 min; *p* = 0.047); underwent lymph-nodal dissection more frequently (15.2% vs. 8.3%; *p* = 0.010); had malignant disease at final pathology (13.6% vs. 7.9%; *p* = 0.017); had lower PTH levels (4.4 vs. 6.0 pg/mL; *p* < 0.001), lower serum ionized calcium levels (1.03 vs. 1.06 mmol/L; *p* < 0.001), and lower serum total albumin-corrected calcium levels on the 1st POD (1.94 vs. 2.02 mmol/L; *p* < 0.001); more frequently underwent intravenous calcium administration at the first or second POD (29.4% vs. 9.3%; *p* = 0.004) and had higher dose of calcium/active vitamin D analogue therapy at discharge (20% vs. 8.2% vs. 0.7%; *p* < 0.001); and underwent follow-up in group B (32.8% vs. 2.4%; *p* < 0.001) and postoperative radioiodine therapy (15.2% vs. 8.6%; *p* = 0.043), while no significant differences occurred for age, gender, arterial hypertension, diabetes and glucose intolerance, preoperative serum vitamin D and PTH levels, hyperthyroidism, laboratory and histological autoimmune thyroiditis, PGRIS, parathyroid reimplantation, reoperations, thyroid weight, and first POD serum phosphorus levels. At stepwise analysis, only follow-up in group B (HR: 2.470; *p* < 0.001) and a higher dose of calcium/active vitamin D analogue therapy at discharge (HR: 0.656, *p* < 0.001) were independent risk factors for persistent HypoPTH.

**Figure 1:**
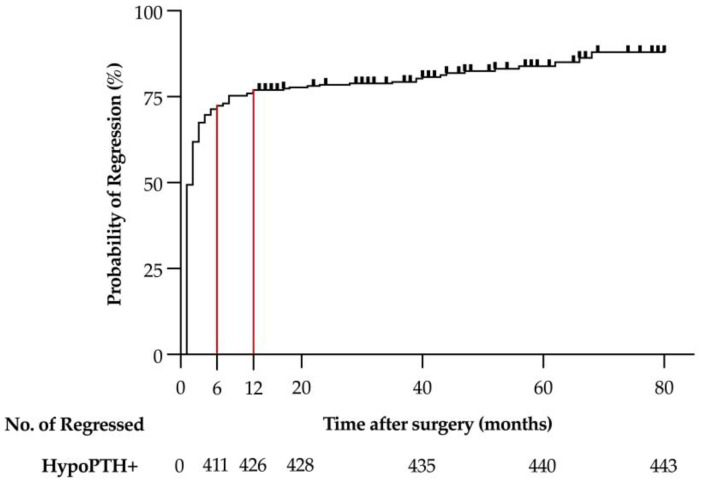
Regression of postoperative hypoparathyroidism in a series of 496 patients after bilateral thyroid surgery.

The median time to HypoPTH regression after surgery was 1 month (range 0–68); regression occurred within 6 months in 411 patients (93%), between 6 and 12 months in 11 patients (2%), and after 1 year in 21 patients (5%) (Figure 1). 

**Figure 2:**
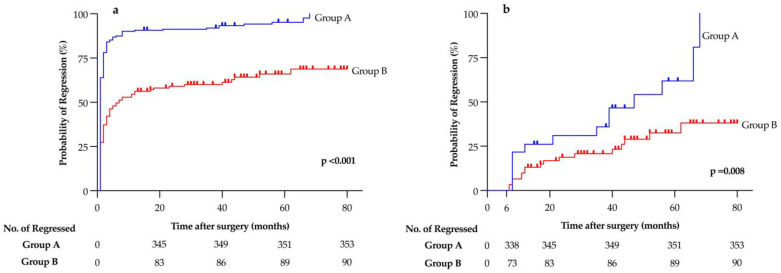
Regression of postoperative hypoparathyroidism in patients undergoing follow-up by endocrine surgeons (Group A) and other physicians (Group B) (**a**); regression in patients with hypoparathyroidism lasting more than 6 months after surgery (**b**).

The median time to HypoPTH regression was significantly lower in patients who underwent follow-up in group A (1 vs. 6 months; *p* < 0.001) that also had higher HypoPTH recovery rates *(*Figure 2a); moreover, considering only patients with persistent HypoPTH lasting more than 6 months after surgery, regression was still significantly more frequent in group A (*p* = 0.008) (Figure 2b).

**Figure 3:**
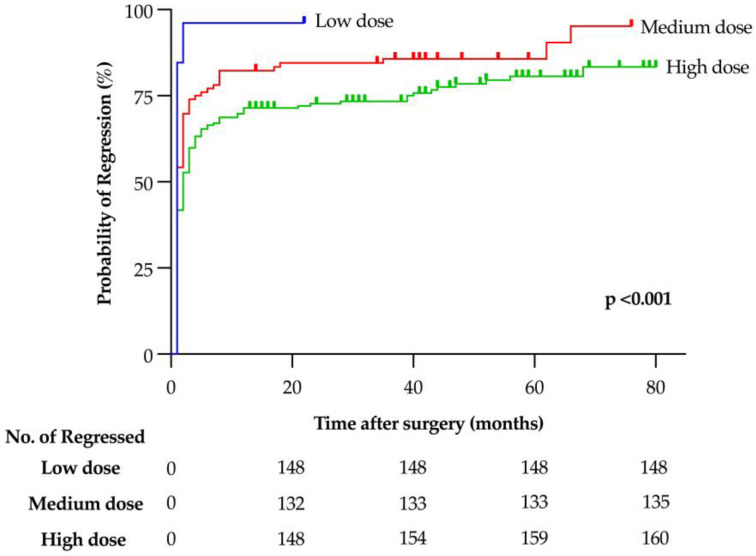
Regression of postoperative hypoparathyroidism according to the dose of calcium/active vitamin D treatment at discharge.

Patients with HypoPTH who received a lower dose of calcium/active vitamin D at discharge had a significantly higher probability of regression (*p* < 0.001) (Figure 3*)*.

## 4. Discussion

HypoPTH is the most frequent morbidity after total thyroidectomy [1,2,3,4]; it is usually reported with variable prevalence, but it represents a relevant problem even in high-volume centers (27.6% and 2.7% in the present series for transient and persistent variants of HypoPTH, respectively).

Moreover, there is no agreement about the definition of postoperative HypoPTH; in some studies, it has been defined according to postoperative hypocalcemia, with or without the presence of related symptoms, even though the most accurate and sensitive definition is postoperative low PTH level [4,26], as used in the present study. Thus, the results reported in the literature are not easily comparable. Several predictive and risk factors have been reported; the identification of the former (related to the immediate postoperative biochemical features) has pivotal relevance to identifying patients needing early treatment before they become symptomatic; the latter are relevant for lowering and possibly eliminating the risk of permanent HypoPTH. 

Some of the risk factors of HypoPTH are related to the patient or to the thyroid disease and subsequently cannot be modified. In the present series, younger age at surgery was found to be a risk factor, in contrast with other studies that identified older age, possibly due to vitamin D deficiency with subsequent deficit in intestinal calcium absorption, as occurs in elderly individuals [12]. In the present series, vitamin D, gender, BMI, and hyperthyroidism were not significant risk factors, in agreement with a recent meta-analysis that highlighted that anamnestic factors cannot predict HypoPTH [11].

In the present series, preoperative lower PTH levels proved to be an independent risk factor of HypoPTH, but this may be related to the definition itself of HypoPTH, since lower baseline PTH levels can lead more easily to a further decrease after surgery, beyond the threshold used to define HypoPTH.

Also, the histological diagnosis of malignancy and the presence of thyroiditis were relevant risk factors for transient HypoPTH (instead, histological thyroiditis was not a risk factor for persistent HypoPTH), probably because they were related to more challenging surgical procedures. In fact, a longer duration of surgery, lymph-nodal dissection, parathyroid reimplantation, and a lower number of PGRIS appeared to be important independent risk factors. Lymph-nodal excision may increase the probability of accidental removal of parathyroid glands and the need for autotransplantation, leading to lower PGRIS [8,17,24]. Thus, the number of PGRIS remains the main independent risk factor; surgery should be aimed at preserving, as much as possible, the parathyroid glands in situ, possibly avoiding systematic parathyroid reimplantation, as previously suggested [25], and limiting prophylactic lymph-nodal removal when not clearly needed [8]. Moreover, new techniques such as fluorescence imaging have been seen to be a useful tool to avoid accidental extirpation of parathyroid glands, especially in surgery for malignancy, when a central lymph-nodal dissection is often required [27].

However, most HypoPTH may regress early, even within the first postoperative month, and usually within 6 months. The present series revealed that HypoPTH may recover even beyond 6 and 12 months after surgery (7% and 5%, respectively), and, in some cases, even later (up to more than 5 years). Thus, the usually reported cut-off of 6–12 months used to define permanent HypoPTH [23] should be used with caution; for this reason, the term “persistent HypoPTH” was considered preferable in the present study.

Persistent HypoPTH remains the main source of concern after bilateral thyroid surgery, since it may seriously impact quality of life and even survival [1,2,3,4,5,28]; surgery should be aimed at minimizing the rate of such morbidity. It cannot be reliably predicted at discharge, even if lower serum calcium and PTH on the first POD were usually present in case of persistent HypoPTH. 

In addition to duration of surgery, lymph-nodal removal, and diagnosis of malignancy at definitive histology, radioactive iodine administration after surgery, the need for intravenous calcium administration on the first and/or second POD, the dose of administered calcium/active vitamin D analogues, and the type of follow-up were relevant risk factors for persistence in the present series. 

Very few studies have focused on the harmful role of radioactive iodine therapy on parathyroid glands [29]; radioactivity may damage the parathyroid cells directly (as may occur after external beam irradiation) or indirectly following the uptake of radioactive Iodine in the thyroid remnants. This finding seems to be confirmed by the present study, although radioiodine was not an independent risk factor, similar to the need for intravenous calcium administration, a possible indirect sign of acute and severe symptomatic hypocalcemia.

Interestingly, the only independent risk factors for persistent HypoPTH were the administration of postoperative higher doses of calcium/active vitamin D analogues and a follow-up not being performed by the operating endocrine surgical team. 

In fact, patients who received a substitutive lower dose of calcium/active vitamin D at discharge regressed sooner and more often than patients receiving higher doses. However, it is not possible to assess whether patients receiving higher doses had more severe HypoPTH, requiring a more aggressive substitutive treatment to remain normocalcemic, or whether higher doses inhibited the recovery of PTH secretion. Recent studies have even suggested that higher doses may “splint” the parathyroid function after surgery, facilitating the recovery of parathyroid function [8,9]. However, the finding of the present study refers only to the dose at discharge, and does not consider eventual modification during the follow-up, impairing the value of any definitive conclusion.

Interestingly, the most relevant independent protective factor against persistent HypoPTH was a follow-up directly performed by the team of operating endocrine surgeons. To the best of our knowledge, this is the first study focusing on the type of physician performing the follow-up in HypoPTH patients.

Indeed, HypoPTH seemed to regress more often in patients undergoing the follow-up by endocrine surgeons compared to patients followed up by other physicians (i.e., general practitioners or other referral physicians). This finding could be due to a tailored and prolonged follow-up, aimed at tapering and lowering the dose of the treatment according to calcium and PTH levels until the withdrawal, with a possibly concomitant progressive stimulation of the parathyroid function. Moreover, although this strategy is time- and resource-consuming, since it needs a personalized and prolonged follow-up, it may lead to avoiding a potentially unnecessary lifelong substitutive treatment in patients with unrecognized and recovered parathyroid function. This finding was even more evident in patients with HypoPTH lasting more than 6 months, in which parathyroid insufficiency is usually considered as definitive. In fact, the current guidelines state that PTH measurement is not even necessary once definitive HypoPTH is diagnosed [30], leading to a prolonged substitutive treatment that cannot be stopped without the confirmation of normal and adequate PTH levels. In patients with an underestimated recovery of the parathyroid function, unnecessary treatment with calcium and active vitamin D analogues may lead to hypercalcemic crisis and urolithiasis.

Therefore, the present study emphasizes the relevance of a prolonged and tailored follow-up, measuring both calcium and PTH levels and trying to lower, taper, and withdraw the treatment, since a regression is possible even in the long-term after surgery.

The present study has some limitations, mainly due to its retrospective nature, which does not allow complete data accuracy. It was conducted in a tertiary, high-volume center, which may not reflect the epidemiology of the disease in the “real world”, since some risk factors (such as reoperations and hyperthyroidism) previously reported in other series [16,19,20,22,25] were not significant predictors of HypoPTH in the present one.

Moreover, even if low PTH levels are considered the most accurate definition of HypoPTH, leading to substitutive treatment, it was evident in the present series that some patients discontinued the calcium/Vitamin D treatment very early after surgery (even within the first week), possibly suggesting that no substitutive treatment might have been really needed. Thus, the effective rate of HypoPTH may have been overestimated according to the PTH cut-off used in the present study. Probably, symptomatic hypocalcemia associated with low PTH levels might represent an alternative endpoint in defining postoperative HypoPTH, but it should be validated only by prospective studies.

## 5. Conclusions

HypoPTH is one of the main factors of morbidity following thyroid surgery. Independent risk factors for transient HypoPTH include a central lymph-nodal dissection for malignancy, lower standardized preoperative PTH levels, longer surgery duration, and fewer PGRIS. A lower dose of calcium and vitamin D therapy at discharge and a follow-up performed by the operating endocrine surgeons are independent protective factors for permanent HypoPTH.

## Figures and Tables

**Table 1:** Risk of HypoPTH after bilateral thyroid surgery in a series of 1965 patients.

		Univariate Logistic Regression Analysis					Stepwise Logistic Regression Analysis		
		HypoPTH− *n* = 1423	HypoPTH+ *n* = 542	Odds Ratio	95% CI	*p* Value	Odds Ratio	95% CI	*p* Value
Age at surgery (years) *		53.14 (14.92)	50.60 (15.98)	0.989	0.983–0.996	0.001	-	-	-
Gender	Female *n* = 1522	1088 (71.2)	434 (28.5)	1.240	0.970–1.58	0.087	-	-	-
	Male *n* = 443	335 (75.6)	108 (24.4)						
Arterial Hypertension	Yes *n* = 553	411 (74.3)	142 (25.7)	0.874	0.699–1.090	0.237	-	-	-
	No *n* = 1412	1012 (71.7)	400 (28.3)						
Body mass index (Kg/m^2^) °		25.06 (5.70)	24.15 (3.96)	0.978	0.953–1.000	0.091	-	-	-
Diabetes and Glucose intolerance	Yes *n* = 126	92 (73.0)	34 (27.0)	0.968	0.645–1.450	0.877	-	-	-
	No *n* = 1839	1331 (72.4)	508 (27.6)						
Preoperative Vitamin D	Decreased *n* = 256	189 (73.8)	67 (26.2)	1.290	0.822–2.030	0.266	-	-	-
	Normal *n* = 140	96 (68.6)	44 (31.4)						
Standardized preoperative PTH °		0.77 (0.38)	0.65 (0.36)	0.293	0.172–0.498	<0.001	3.190	1.860–5.470	<0.001
Preoperative serum total albumin-corrected calcium levels (mmol/L) *		2.33 (0.12)	2.33 (0.13)	1.350	0.587–3.090	0.482	-	-	-
Hyperthyroidism	Yes *n* = 503	380 (75.5)	123 (24.5)	0.806	0.638–1.020	0.069	-	-	-
	No *n* = 1462	1043 (71.3)	419 (28.7)						
Laboratory Autoimmune Thyroiditis	Yes *n* = 766	556 (72.6)	210 (27.4)	0.971	0.787–1.200	0.787	-	-	-
	No *n* = 993	715 (72.0)	278 (28.0)						
Surgery duration (minutes) °		75 (25)	80 (30)	1.000	1.000–1.010	0.002	1.010	1.000–1.021	0.032
Lymph-nodal dissection	Yes *n* = 503	316 (62.8)	187 (37.2)	1.850	1.490–2.290	<0.001	1.550	1.090–2.190	0.015
	No *n* = 1462	1107 (75.7)	355 (24.3)						
PGRIS	≤2 *n* = 61	19 (31.1)	42 (68.9)	0.162	0.093–0.281	<0.001	5.050	2.340–10.900	<0.001
	>2 *n* = 1889	1390 (73.6)	499 (26.4)						
Parathyroid reimplantation	Yes *n* = 92	49 (53.3)	43 (46.7)	2.420	1.580–3.690	<0.001	-	-	-
	No *n* = 1873	1374 (73.4)	499 (26.6)						
Reoperations	Yes *n* = 36	30 (83.3)	6 (16.7)	0.520	0.215–1.260	0.146	-	-	-
	No *n* = 1929	1393 (72.2)	536 (27.8)						
Histological diagnosis	Benign *n* = 1122	840 (74.9)	282 (25.1)	1.330	1.090–1.620	0.005	-	-	-
	Malignant *n* = 843	583 (69.2)	260 (30.8)						
Histological Autoimmune Thyroiditis	Yes *n* = 357	234 (65.5)	123 (34.5)	1.480	1.160–1.890	0.002	-	-	-
	No *n* = 1594	1176 (73.8)	418 (26.2)						
Thyroid weight (grams) °		31.00 (44.40)	33.00 (42.25)	1.000	0.999–1.000	0.311	-	-	-

Abbreviations: HypoPTH+ (patients with postoperative HypoPTH), HypoPTH− (patients without postoperative HypoPTH), PGRIS (parathyroid glands remained in situ), 95% CI (95% confidence interval). Values in parentheses are percentages unless indicated otherwise; values are: * mean (s.d.) and ° median (interquartile range). Data available for BMI (1401 patients, 71.3%), preoperative vitamin D levels (396 patients, 20.2%), preoperative PTH levels (972 patients, 49.5%), preoperative serum total albumin-corrected calcium levels (1948 patients, 99.1%), laboratory autoimmune thyroiditis (1759 patients, 89.5%), PGRIS (1950 patients, 99.2%), surgery duration (1954 patients, 99.4%), thyroid weight (1946 patients, 99.0%), and histological thyroiditis (1951 patients, 99.3%).

**Table 2:** Risk and predictive factors of persistent HypoPTH in a series of 496 patients after bilateral thyroid surgery.

		Univariate Cox Proportional Regression Analysis					Stepwise Cox Proportional Regression Analysis		
		Regressed HypoPTH *n* = 443	Persistent HypoPTH *n* = 53	Hazard Ratio	95% CI	*p* Value	Hazard Ratio	95% CI	*p* Value
Age at surgery (years) *		50.77 (16.01)	48.73 (16.53)	1.005	0.999–1.011	0.113	-	-	-
Gender	Female *n* = 404	363 (89.9)	41 (10.1)	0.960	0.754–1.224	0.744	-	-	-
	Male *n* = 92	80 (87.0)	12 (13.0)						
Arterial Hypertension	Yes *n* = 126	117 (92.9)	9 (7.1)	1.178	0.954–1.456	0.128	-	-	-
	No *n* = 370	326 (88.1)	44 (11.9)						
Body mass index (Kg/m^2^) °		24.19 (6.07)	23.31 (5.82)	1.009	0.986–1.033	0.456	-	-	-
Diabetes and Glucose intolerance	Yes *n* = 32	29 (90.6)	3 (9.4)	1.001	0.687–1.460	0.994	-	-	-
	No *n* = 464	414 (89.2)	50 (10.8)						
Preoperative Vitamin D	Decreased *n* = 65	55 (84.6)	10 (15.4)	1.315	0.871–1.986	0.192	-	-	-
	Normal *n* = 42	40 (95.2)	2 (4.8)						
Standardized preoperative PTH °		0.66 (0.38)	0.61 (0.32)	0.960	0.608–1.517	0.862	-	-	-
Hyperthyroidism	Yes *n* = 109	100 (91.7)	9 (8.3)	0.999	0.800–1.249	0.995	-	-	-
	No *n* = 387	343 (88.6)	44 (11.4)						
Laboratory Autoimmune Thyroiditis	Yes *n* = 187	163 (87.2)	24 (12.8)	0.873	0.715–1.066	0.182	-	-	-
	No *n* = 259	236 (91.1)	23 (8.9)						
Surgery duration (minutes) °		80 (30)	85 (35)	0.997	0.994–1.000	0.047	-	-	-
Lymph-nodal dissection	Yes *n* = 171	145 (84.8)	26 (15.2)	0.768	0.628–0.938	0.010	-	-	-
	No *n* = 325	298 (91.7)	27 (8.3)						
PGRIS	≤2 *n* = 40	34 (85.0)	6 (15.0)	1.105	0.779–1.569	0.575	-	-	-
	>2 *n* = 455	408 (89.7)	47 (10.3)						
Parathyroid glands reimplantation	Yes *n* = 38	35 (92.1)	3 (7.9)	0.991	0.702–1.400	0.961	-	-	-
	No *n* = 458	408 (89.0)	50 (11.0)						
Reoperations	Yes *n* = 6	5 (83.3)	1 (16.7)	0.917	0.380–2.214	0.847	-	-	-
	No *n* = 490	438 (89.4)	52 (10.6)						
Histological diagnosis	Benign *n* = 254	234 (92.1)	20 (7.9)	0.796	0.660–0.960	0.017	-	-	-
	Malignant *n* = 242	209 (86.4)	33 (13.6)						
Histological Autoimmune Thyroiditis	Yes *n* = 114	103 (90.4)	11 (9.6)	1.002	0.804–1.250	0.984	-	-	-
	No *n* = 381	339 (89.0)	42 (11.0)						
Thyroid weight (grams) °		32.50 (43.00)	34.00 (24.00)	1.000	0.999–1.001	0.690	-	-	-
1st POD PTH levels (pg/mL) °		6.0 (3.6)	4.4 (1.4)	1.218	1.160–1.279	<0.001	-	-	-
1st POD serum total albumin-corrected calcium levels (mmol/L) *		2.02 (0.15)	1.94 (0.14)	5.362	2.870–10.020	<0.001	-	-	-
1st POD serum ionized calcium levels (mmol/L) °		1.06 (0.07)	1.03 (0.09)	154.800	32.250–743.300	<0.001	-	-	-
1st POD phosphorus levels (mmol/L) °		1.46 (0.25)	1.48 (0.27)	0.739	0.514–1.062	0.102	-	-	-
Calcium intravenous administration in 1st or 2nd POD	Yes *n* = 34	24 (70.6)	10 (29.4)	0.548	0.362–0.828	0.004	-	-	-
	No *n* = 462	419 (90.7)	43 (9.3)						
Calcium/Vitamin D treatment at discharge	Low dose *n* = 149	148 (99.3)	1 (0.7)	0.512	0.450–0.582	<0.001	0.656	0.580–0.743	<0.001
	Medium dose *n* = 147	135 (91.8)	12 (8.2)						
	High dose *n* = 200	160 (80.0)	40 (20.0)						
Physicians performing the follow-up	Group A *n* = 362	353 (97.5)	9 (2.5)	2.949	2.313–3.760	<0.001	2.470	1.919–3.178	<0.001
	Group B *n* = 134	90 (67.2)	44 (32.8)						
RadioIodine therapy	Yes *n* = 158	134 (84.8)	24 (15.2)	0.810	0.661–0.994	0.043			
	No *n* = 338	309 (91.4)	29 (8.6)						

Abbreviations: PGRIS (parathyroid glands remained in situ), POD (first postoperative day); 95% CI (95% confidence interval). Group A: endocrine surgeons; Group B: other physicians. Values in parentheses are percentages unless indicated otherwise; values are: * mean (s.d.) and ° median (q.r.i). Data available for body mass index (349 patients, 70.4%), preoperative Vitamin D levels (107 patients, 21.6%), preoperative PTH levels (233 patients, 47.0%), laboratory autoimmune thyroiditis (446 patients, 89.9%), PGRIS (495 patients, 99.8%), surgery duration (494 patients, 99.6%), thyroid weight (493 patients, 99.4%), histological thyroiditis (495 patients, 99.8%), 1st POD PTH levels (481 patients, 97.0%), 1st POD serum ionized calcium levels (484 patients, 97.6%), and 1st POD phosphorus levels (494 patients, 99.6%).

## Data Availability

The raw data supporting the conclusions of this article will be made available by the authors on request.

## References

[1] Ru Z., Mingliang W., Maofei W., Qiaofeng C., Jianming Y. (2021). Analysis of Risk Factors for Hypoparathyroidism after Total Thyroidectomy. Front. Surg..

[2] Lončar I., van Kinschot C.M.J., van Dijk S.P.J., Franssen G.J.H., Visser E.E., Peeters R.P., van Eijck C.J.H., van Noord C., van Ginhoven T.M. (2022). Persistent Post-Thyroidectomy Hypoparathyroidism: A Multicenter Retrospective Cohort Study. Scand. J. Surg..

[3] Takahashi T., Yamazaki K., Shodo R., Ueki Y., Horii A. (2022). Actual Prevalence of Hypoparathyroidism after Total Thyroidectomy: A Health Insurance Claims-Database Study. Endocrine.

[4] Nagel K., Hendricks A., Lenschow C., Meir M., Hahner S., Fassnacht M., Wiegering A., Germer C.-T., Schlegel N. (2022). Definition and Diagnosis of Postsurgical Hypoparathyroidism after Thyroid Surgery: Meta-Analysis. BJS Open.

[5] Marcucci G., Cianferotti L., Parri S., Altieri P., Arvat E., Benvenga S., Betterle C., Bondanelli M., Boscaro M., Camozzi V. (2018). HypoparaNet: A Database of Chronic Hypoparathyroidism Based on Expert Medical-Surgical Centers in Italy. Calcif. Tissue Int..

[6] Harsløf T., Rolighed L., Rejnmark L. (2019). Huge Variations in Definition and Reported Incidence of Postsurgical Hypoparathyroidism: A Systematic Review. Endocrine.

[7] Orloff L.A., Wiseman S.M., Bernet V.J., Fahey T.J., Shaha A.R., Shindo M.L., Snyder S.K., Stack B.C., Sunwoo J.B., Wang M.B. (2018). American Thyroid Association Statement on Postoperative Hypoparathyroidism: Diagnosis, Prevention, and Management in Adults. Thyroid.

[8] Sitges-Serra A. (2017). The PGRIS and Parathyroid Splinting Concepts for the Analysis and Prognosis of Protracted Hypoparathyroidism. Gland Surg..

[9] Sitges-Serra A., Gómez J., Barczynski M., Lorente-Poch L., Iacobone M., Sancho J. (2017). A Nomogram to Predict the Likelihood of Permanent Hypoparathyroidism after Total Thyroidectomy Based on Delayed Serum Calcium and iPTH Measurements. Gland Surg..

[10] Sitges-Serra A., Ruiz S., Girvent M., Manjón H., Dueñas J.P., Sancho J.J. (2010). Outcome of Protracted Hypoparathyroidism after Total Thyroidectomy. Br. J. Surg..

[11] Edafe O., Antakia R., Laskar N., Uttley L., Balasubramanian S.P. (2014). Systematic Review and Meta-Analysis of Predictors of Post-Thyroidectomy Hypocalcaemia. Br. J. Surg..

[12] Erbil Y., Barbaros U., Temel B., Turkoglu U., Işsever H., Bozbora A., Ozarmağan S., Tezelman S. (2009). The Impact of Age, Vitamin D(3) Level, and Incidental Parathyroidectomy on Postoperative Hypocalcemia after Total or near Total Thyroidectomy. Am. J. Surg..

[13] Kamer E., Unalp H.R., Erbil Y., Akguner T., Issever H., Tarcan E. (2009). Early Prediction of Hypocalcemia after Thyroidectomy by Parathormone Measurement in Surgical Site Irrigation Fluid. Int. J. Surg..

[14] Erbil Y., Barbaros U., Ozbey N., Aral F., Ozarmağan S. (2009). Risk Factors of Incidental Parathyroidectomy after Thyroidectomy for Benign Thyroid Disorders. Int. J. Surg..

[15] Lindblom P., Westerdahl J., Bergenfelz A. (2002). Low Parathyroid Hormone Levels after Thyroid Surgery: A Feasible Predictor of Hypocalcemia. Surgery.

[16] Promberger R., Ott J., Kober F., Mikola B., Karik M., Freissmuth M., Hermann M. (2010). Intra- and Postoperative Parathyroid Hormone-Kinetics Do Not Advocate for Autotransplantation of Discolored Parathyroid Glands during Thyroidectomy. Thyroid.

[17] Baud G., Jannin A., Marciniak C., Chevalier B., Do Cao C., Leteurtre E., Beron A., Lion G., Boury S., Aubert S. (2022). Impact of Lymph Node Dissection on Postoperative Complications of Total Thyroidectomy in Patients with Thyroid Carcinoma. Cancers.

[18] van Beek D.-J., Almquist M., Bergenfelz A.O., Musholt T.J., Nordenström E., the EUROCRINE^®^ Council (2021). Complications after Medullary Thyroid Carcinoma Surgery: Multicentre Study of the SQRTPA and EUROCRINE^®^ Databases. Br. J. Surg..

[19] Applewhite M.K., White M.G., Xiong M., Pasternak J.D., Abdulrasool L., Ogawa L., Suh I., Gosnell J.E., Kaplan E.L., Duh Q.-Y. (2016). Incidence, Risk Factors, and Clinical Outcomes of Incidental Parathyroidectomy During Thyroid Surgery. Ann. Surg. Oncol..

[20] Sitges-Serra A., Gallego-Otaegui L., Suárez S., Lorente-Poch L., Munné A., Sancho J.J. (2017). Inadvertent Parathyroidectomy during Total Thyroidectomy and Central Neck Dissection for Papillary Thyroid Carcinoma. Surgery.

[21] Lorenz K., Raffaeli M., Barczyński M., Lorente-Poch L., Sancho J. (2020). Volume, Outcomes, and Quality Standards in Thyroid Surgery: An Evidence-Based Analysis—European Society of Endocrine Surgeons (ESES) Positional Statement. Langenbecks Arch. Surg..

[22] Sosa J.A., Bowman H.M., Tielsch J.M., Powe N.R., Gordon T.A., Udelsman R. (1998). The Importance of Surgeon Experience for Clinical and Economic Outcomes from Thyroidectomy. Ann. Surg..

[23] Pasieka J.L., Wentworth K., Yeo C.T., Cremers S., Dempster D., Fukumoto S., Goswami R., Houillier P., Levine M.A., Pasternak J.D. (2022). Etiology and Pathophysiology of Hypoparathyroidism: A Narrative Review. J. Bone Miner. Res..

[24] Sitges-Serra A. (2021). Etiology and Diagnosis of Permanent Hypoparathyroidism after Total Thyroidectomy. J. Clin. Med..

[25] Lorente-Poch L., Sancho J.J., Ruiz S., Sitges-Serra A. (2015). Importance of in Situ Preservation of Parathyroid Glands during Total Thyroidectomy. Br. J. Surg..

[26] Selberherr A., Scheuba C., Riss P., Niederle B. (2015). Postoperative Hypoparathyroidism after Thyroidectomy: Efficient and Cost-Effective Diagnosis and Treatment. Surgery.

[27] Demarchi M.S., Seeliger B., Lifante J.-C., Alesina P.F., Triponez F. (2021). Fluorescence Image-Guided Surgery for Thyroid Cancer: Utility for Preventing Hypoparathyroidism. Cancers.

[28] Bergenfelz A., Nordenström E., Almquist M. (2020). Morbidity in Patients with Permanent Hypoparathyroidism after Total Thyroidectomy. Surgery.

[29] Abuduwaili M., Baidula W., Xia B., Wu Z., Chen Z., Xing Z., Su A. (2022). The Effects of Radioiodine Therapy on the Recovery of Parathyroid Function in Patients with Protracted Hypoparathyroidism after Total Thyroidectomy for Papillary Thyroid Carcinoma. J. Investig. Surg..

[30] Bollerslev J., Rejnmark L., Zahn A., Heck A., Appelman-Dijkstra N.M., Cardoso L., Hannan F.M., Cetani F., Sikjær T., Formenti A.M. (2021). European Expert Consensus on Practical Management of Specific Aspects of Parathyroid Disorders in Adults and in Pregnancy: Recommendations of the ESE Educational Program of Parathyroid Disorders. Eur. J. Endocrinol..

